# Brain metabolic resilience conferred by the APOE3 Christchurch variant in autosomal dominant Alzheimer’s disease and across the adult lifespan

**DOI:** 10.21203/rs.3.rs-10145165/v1

**Published:** 2026-08-14

**Authors:** Yakeel Quiroz, Catarina Tristão-Pereira, Vincent Malotaux, Yesica Zuluaga, Claudia Muñoz, George Silva, Bing He, Diana Alzate, Yamile Bocanegra, Alejandro Guerrero, Laura Serna, Carolina Ospina, Monica Vidal, Sergio Alvarez, Rafael Posada-Duque, Joseph Arboleda-Velasquez, David Aguillon

**Affiliations:** Massachusetts General Hospital, Harvard Medical School; Boston University; Boston University; Grupo de Neurociencias de Antioquia, Facultad de Medicina, Universidad de Antioquia, Medellín, Colombia; Grupo de Neurociencias de Antioquia; Boston University; Boston University; Grupo de Neurociencias de Antioquia, Facultad de Medicina, Universidad de Antioquia, Medellín, Colombia; Universidad de Antioquia; Universidad de Antioquia; Universidad de Antioquia; Universidad de Antioquia; Hospital Pablo Tobón Uribe; Hospital Pablo Tobón Uribe; Universidad de Antioquia; Schepens Eye Research Institute of Mass Eye and Ear and Department of Ophthalmology, Harvard Medical School, Boston, USA; Universidad de Antioquia

## Abstract

The *APOE3* Christchurch (*APOE3^Ch^*) is a rare genetic variant associated with marked protection against autosomal-dominant Alzheimer’s disease (ADAD) due to the *PSEN1^E280A^* variant, by delaying the onset of clinical symptoms. In this study, we investigate age-related alterations in cerebral glucose metabolism, measured with 18F-fluorodeoxyglucose positron emission tomography, and cognitive performance in: 1) *APOE3^Ch^*-*PSEN1^E280A^* carriers (n = 7) compared to typical *PSEN1^E280A^* carriers (n = 48) across the ADAD continuum; and 2) cognitively-unimpaired *APOE3^Ch^* carriers (n = 29) compared to non-carriers (n = 43) across the adult lifespan. Across ADAD, *APOE3^Ch^*-*PSEN1^E280A^* carriers had a 58% smaller age-related reduction in glucose metabolism in the precuneus, along with a 50% smaller reduction in episodic memory, compared to typical *PSEN1^E280A^* carriers. Across the adult lifespan, *APOE3^Ch^* carriers had remarkably preserved glucose metabolism in the anterior cingulate cortex, compared to non-carriers (slope: 0.001 versus – 0.009 /year). In this region, higher glucose metabolism in *APOE3^Ch^* carriers correlated with greater global cognition and episodic memory, as well as with fewer subjective memory complaints and depressive symptoms. Our findings suggest that the *APOE3^Ch^* variant may be protective against brain metabolic changes both in ADAD and across the disease-free adult lifespan, with corresponding benefits in cognitive function. Understanding the pathways through which *APOE3^Ch^* confers metabolic protective effects could inform novel therapeutic avenues to enhance resilience against AD and promote healthy brain aging.

## Introduction

The *APOE3* Christchurch (*APOE3^Ch^*) is a rare variant (R136S) that may protect against neurodegeneration and Alzheimer’s disease (AD). The first evidence of this protection was demonstrated in autosomal dominant AD (ADAD) due to the *PSEN1^E280A^* genetic variant from the Colombian ADAD kindred. In typical *PSEN1^E280A^* carriers, the onset of mild cognitive impairment (MCI) occurs by age 44 and the onset of dementia by age 49^1^. The disease trajectory is characterized by progressive amyloid-β (Aβ) accumulation beginning approximately 20 years before the onset of cognitive symptoms, followed by tau pathology, cerebral glucose hypometabolism and neuronal injury^2,3^, largely mirroring the late-onset AD progression^4^.

Despite the near-complete penetrance of this *PSEN1* variant, the homozygous *APOE3^Ch^* carrier found in the Colombian kindred developed MCI about 30 years after the expected age of onset^5^. Notably, at age 75, this individual had a high burden of Aβ but limited tau pathology and preserved cerebral glucose metabolism. Within the kindred, 27 *PSEN1^E280A^* carriers were also found to be heterozygous for *APOE3^Ch^* and showed a 5-year delay in cognitive decline, suggesting that the *APOE3^Ch^* variant may confer a dose-dependent protection against ADAD^6^. Imaging outcomes were similar in individuals with one copy of the *APOE3^Ch^* variant compared to the case carrying two copies, consistent with mechanisms that reduce tau pathology and neurodegeneration, even in the presence of high Aβ burden^7^. However, imaging results derive from samples of only one or two individuals and larger sample sizes are warranted to elucidate *APOE3^Ch^*-related protective phenotypes.

While protective in ADAD, whether the *APOE3^Ch^* variant may confer protection against age-related decline in individuals without the *PSEN1* variant remains unknown. Across the adult lifespan, cerebral glucose metabolism, measured by 18F-fluorodeoxyglucose (FDG) positron emission tomography (PET), has been shown to decline with age, even in the absence of overt pathology^8,9^. In addition, regional hypometabolism precedes and predicts subtle cognitive changes in healthy older adults, prior to cognitive impairment^10-12^. These findings support the utility of FDG-PET as a sensitive biomarker to gradual age-related changes and its potential to evaluate *APOE3^Ch^*-related protective effects against hypometabolism.

Therefore, this study investigated age-related alterations in cerebral glucose metabolism and in 1) heterozygous *APOE3^Ch^*-*PSEN1^E280A^* carriers compared to typical *PSEN1^E280A^* carriers across the ADAD continuum; and in 2) cognitively unimpaired heterozygous *APOE3^Ch^* carriers compared to non-*PSEN1^E280A^* carriers across the adult lifespan. We hypothesized that the *APOE3^Ch^* variant would provide brain metabolic protection, such that both dual *APOE3^Ch^*-*PSEN1^E280A^* and *APOE3^Ch^* carriers would show greater preservation of cerebral glucose metabolism with age compared to their respective control groups. Additionally, we examined associations between cerebral glucose metabolism and cognitive performance across groups. Given the rarity of the *APOE3^Ch^* variant, with a 0.004% allele frequency in a UK Biobank sample of 455,306 individuals^13^ and a 0.003% global frequency in gnomAD, the Colombian kindred represents a unique opportunity to study its biological and clinical significance.

## Results

### Study demographics

Among *PSEN1^E280A^* carriers, 48 were non-carriers for *APOE3^Ch^* (M_age_: 39 [32, 45] years, 63% females, 35% cognitively impaired) and 7 were heterozygous for *APOE3^Ch^* (M_age_: 49 [35, 53], 29% females, 29% cognitively impaired) (Table 1). There were no statistically significant differences in demographics or cognitive metrics between groups, possibly due to imbalanced and small sample sizes. Among cognitively unimpaired non-*PSEN1^E280A^* carriers, 43 were non-carriers for *APOE3^Ch^* (M_age_: 40 [36, 44] years, 60% females) and 29 were heterozygous for *APOE3^Ch^* (M_age_: 43 [35, 50], 52% females). *APOE3^Ch^* carriers had less years of education (11 [5, 13] versus 12 [8, 15] years, *p* = 0.034), lower processing speed (higher Trail Making Test Part A [TMTA] time: 49 [39, 62] versus 40 [30, 50] s, *p* = 0.016) and greater burden of depressive symptoms (higher Geriatric Depressive Scale [GDS] scores: (2 [1, 4] versus 0 [0, 2], *p* = 0.002) than non-carriers. There were no significant differences in age, sex distribution or other cognitive domains.

### Age-related alterations in cerebral glucose metabolism and cognitive performance in *APOE3^Ch^*-*PSEN1^E280A^* carriers across ADAD

To investigate age-related alterations in cerebral glucose metabolism, we first selected *a priori* the precuneus and hippocampus as our regions of interest (ROI), due to their metabolic role in AD pathology and aging^9,14^, and then used an exploratory voxelwise approach. Our adjusted ROI-based analyses within ADAD revealed a significant age-by-group interaction for FDG SUVR in the precuneus (β = 0.011 [0.004, 0.018], *p* = 0.003; Figure 1A), indicating that the association between age and precuneus glucose metabolism differed between groups. Specifically, *APOE3^Ch^*-*PSEN1^E280A^* carriers had a 58% smaller age-related reduction in precuneus glucose metabolism with age (β = −0.008 [−0.012, −0.004]), compared to a steeper reduction in typical *PSEN1^E280A^* carriers (β = −0.019 [−0.027, −0.011]). No significant age-by-group interaction was observed for hippocampal FDG SUVR (β = 0.002 [−0.007, 0.012], *p* = 0.629; Figure 1B). As expected due to our limited sample size, exploratory voxelwise analyses did not survive correction for multiple comparisons. Though FDR-uncorrected clusters should be interpreted with caution, the positive age-by-group contrast suggested a trend for slope differences in the precuneus and frontotemporal regions, consistent with greater preservation of regional glucose metabolism with age in *APOE3^Ch^*-*PSEN1^E280A^* carriers compared to typical *PSEN1^E280A^* carriers (Figure 1C).

Across cognitive performance scores, we examined a battery of neuropsychological tests to assess global cognition, delayed recall, subjective memory complaints and depressive symptoms. There was not a significant age-by-group interaction for global cognition (Mini-Mental State Examination [MMSE] scores: β = 0.129 [−0.098, 0.357], *p* = 0.259; Figure 1D). However, significant slope differences in the Consortium to Establish a Registry for Alzheimer’s disease (CERAD) Word List scores between groups (β = 0.159 [0.050, 0.269], *p* = 0.005; Figure 1E) indicated that delayed recall had a 50% smaller age-related reduction in *APOE3^Ch^*-*PSEN1^E280A^* carriers (β = −0.158 [−0.264, −0.051]) than in typical *PSEN1^E280A^* carriers (β = −0.317 [−0.407, −0.227]). Memory complaints tended to occur later life in *APOE3^Ch^*-*PSEN1^E280A^* carriers than in typical *PSEN1^E280A^* carriers, with similar Subjective Memory Complaints (SMC) scores slopes between groups (β = −0.185 [−0.778, 0.408], *p* = 0.534; Figure 1F). The interaction for GDS scores was also not statistically significant (β = −0.048 [−0.168, 0.072], *p* = 0.426; Figure 1G), but we observed a trend for lower and more stable depressive symptoms with age in *APOE3^Ch^*-*PSEN1^E280A^* carriers compared to typical *PSEN1^E280A^* carriers.

### Age-related alterations in cerebral glucose metabolism and cognitive performance in cognitively unimpaired *APOE3^Ch^* carriers across the adult lifespan

In adjusted ROI-based analysis within the adult lifespan, we did not observe a significant age-by-group interaction for FDG SUVR in the precuneus (β = 0.003 [−0.004, 0.010], *p* = 0.424; Figure 2A). However, there were significant slope differences in hippocampal FDG SUVR (β = 0.003 [0.000, 0.006], *p* = 0.039; Figure 2B), with *APOE3^Ch^* carriers exhibiting a stable hippocampal glucose metabolism over age (β = 0.001 [−0.000, 0.002]), whereas non-carriers for the *APOE3^Ch^* or *PSEN1^E280A^* variants showed a significant age-related reduction in metabolism (β = −0.002 [−0.005, −0.000]). FDR-corrected voxelwise analysis further showed a positive interaction between groups, in a statistical cluster that primarily included the anterior cingulate cortex (ACC; Figure 2C). Post-hoc ROI-based analysis of FDG SUVR in the ACC confirmed an age-by-group interaction (β = 0.008 [0.004, 0.012], *p* < 0.001), in which glucose metabolism did not decrease with age in *APOE3^Ch^* carriers (β = 0.001 [−0.001, 0.002]) in contrast to the age-related reduction in glucose metabolism in non-carriers (β = −0.008 [−0.011, −0.004]). The ACC FDG SUVR model was additionally adjusted for GDS scores and ACC volume and the interaction effect remained significant (β = 0.009 [0.004, 0.013], *p* < 0.001).

As for cognitive performance scores, a significant positive age-by-group interaction for MMSE scores (β = 0.092 [0.009, 0.175], *p* = 0.031; Figure 2D) demonstrated that global cognition decreased with age in non-carriers (β = −0.072 [−0.128, −0.016]) but not in *APOE3^Ch^* carriers (β = −0.020 [−0.046, 0.086]). Though with similar trends as MMSE scores, there were no significant slope differences in delayed recall, subjective memory complaints or depressive symptoms between groups (CERAD Word List: β = 0.032 [−0.069, 0.132], *p* = 0.530; SMC: β = −0.167 [−0.534, 0.201], *p* = 0.368; GDS: β = −0.123 [−0.262, 0.016], *p* = 0.082; Figure 2E-G). There were also no significant differences in secondary cognitive domains over time between groups, including scores assessing language and word retrieval abilities, processing speed and attention, executive function and abstract reasoning (**Supplementary Figure 1A-D**).

### Associations between cerebral glucose metabolism and cognitive performance in cognitively unimpaired *APOE3^Ch^* carriers across the adult lifespan

Given that cerebral glucose metabolism and cognitive performance were stable over time in *APOE3^Ch^* carriers, we computed age-adjusted Z-scores for regional FDG SUVR values and cognitive scores in *APOE3^Ch^* carriers relative to the non-carriers. Adjusted robust regression analyses revealed significant associations between ACC FDG SUVR z-scores and cognitive z-scores, indicating that greater cerebral glucose metabolism in the ACC in *APOE3^Ch^* carriers compared to non-carriers was associated with greater global cognition (MMSE: β = 0.494 [0.074, 0.914], *p* = 0.023; Figure 3A), greater delayed recall (CERAD Word List: β = 0.280 [0.062, 0.497], *p* = 0.014; Figure 3B), lower subjective memory complaints (SMC: β = −0.312 [−0.539, −0.086], *p* = 0.009; Figure 3C) and lower burden of depressive symptoms (GDS: β = −0.312 [−0.620, −0.005], *p* = 0.047; Figure 3D) in *APOE3^Ch^* carriers compared to non-carriers. Greater ACC glucose metabolism in *APOE3^Ch^* carriers compared to non-carriers was not correlated with secondary cognitive domains (**Supplementary Figure 1E-H**). Similar models were not computed across the ADAD population due to the limited sample size.

## Discussion

In this study, we evaluated the effect of the *APOE3^Ch^* variant on age-related changes in cerebral glucose metabolism and cognitive performance in two distinct populations: one group with ADAD due to the *PSEN1^E280A^* variant and one group without the *PSEN1^E280A^* variant representing the normal adult lifespan.

We observed that dual *APOE3^Ch^*-*PSEN1^E280A^* carriers had a smaller age-related reduction in glucose metabolism in the precuneus, accompanied by a smaller age-related reduction in episodic memory, compared to typical *PSEN1^E280A^* carriers. Among cognitively unimpaired *APOE3^Ch^* carriers without the *PSEN1^E280A^* variant, we found a preservation in glucose metabolism in the ACC, compared to non-carriers. This increased glucose metabolism in *APOE3^Ch^* carriers relative to non-carriers correlated with higher global cognition and episodic memory performance and with lower subjective memory complaints and depressive symptoms. Our findings suggest that the *APOE3^Ch^* variant may be protective against brain metabolic changes in the ADAD continuum and across the disease-free lifespan, representing a promising target for enhancing resilience against neurodegenerative diseases.

Within the Colombian kindred, typical *PSEN1^E280A^* carriers exhibit reduced cerebral glucose metabolism approximately 15 years prior to the onset of MCI, affecting primarily the precuneus and posterior cingulate cortex, followed by frontotemporal regions as the disease advances^14^. Here, we compared age-related cerebral glucose metabolism alterations in *PSEN1^E280A^* carriers from the kindred with and without the *APOE3^Ch^* variant. Dual *APOE3^Ch^*-*PSEN1^E280A^* carriers exhibited a 58% slower reduction in glucose metabolism in the precuneus compared to typical *PSEN1^E280A^* carriers (−0.008 versus – 0.019). The regional pattern trending toward smaller glucose metabolism reductions in *APOE3^Ch^*-*PSEN1^E280A^* carriers matched the hypometabolic pattern seen in ADAD and late-onset AD cases^14-16^. These metabolic slope differences were concurrent with a 50% slower rate of episodic memory decline in *APOE3^Ch^*-*PSEN1^E280A^* carriers compared to typical *PSEN1^E280A^* carriers (−0.158 versus – 0.317). Despite the small sample size due to the low frequency of the *APOE3^Ch^* variant, our results support and expand upon the findings from the first *APOE3^Ch^* cases found in the kindred^5,6^.

Limited tau pathology in the presence of high Aβ burden has been proposed to result from increased microglial response to Aβ and reduced *APOE3^Ch^* binding to heparan sulfate proteoglycans, which facilitate tau uptake and propagation^7,17,18^. Considering the temporal cascade of AD, our results suggest that the absence of tau pathology in *APOE3^Ch^*-*PSEN1^E280A^* carriers may hinder the decline in glucose uptake, which would otherwise be near-certain due to the high penetrance of the *PSEN1* variant. Notably, glucose metabolism has been shown to be preserved in response to early Aβ burden in young healthy adults as a neural reserve process^19^. Thus, limited tau pathology and preserved glucose metabolism in *APOE3^Ch^*-*PSEN1^E280A^* carriers may synergistically or independently protect against clinical symptoms, potentially through less severe neurodegeneration, delaying the age of onset. This potential brain metabolic protection conferred by the *APOE3^Ch^* variant against ADAD progression should be interpreted with caution given the limited sample and warrants further investigation. The underlying mechanisms of resilience to disease progression could inform the development of therapeutic approaches, such as the novel disease-modifying *APOE^Ch^*-mimetic antibody^20^.

Less is known about the effect of the *APOE3^Ch^* variant in the absence of deterministic pathology. To our knowledge, this is the first study to explore imaging biomarkers in *APOE3^Ch^* carriers without genetic predisposition to AD. FDG-PET is a highly sensitive biomarker to investigate age-related changes across the disease-free adult lifespan, as cerebral glucose metabolism decreases with healthy aging^8,9^. Indeed, we found that glucose metabolism in the precuneus and hippocampus decreased with age in non-carriers, despite relatively young age. Slope differences between groups were observed in the hippocampus, where glucose metabolism remained preserved with age in *APOE3^Ch^* carriers (0.001 versus – 0.002). Our exploratory analysis further revealed that the ACC was among the most metabolically preserved brain regions in *APOE3^Ch^* carriers (0.001 versus – 0.008). This pattern of metabolic maintenance is notably different than that of dual *APOE3^Ch^*-*PSEN1^E280A^* carriers, suggesting a dissociation between processes involved in resilience to AD-specific pathology versus healthy aging.

In line with our findings, a study on cognitively intact adults over 80 years reported a signature of preserved metabolism that included primarily the ACC^21^. We theorize that genetic protection might act through similar pathways, only decades earlier, though longitudinal follow-up of *APOE3^Ch^* carriers will provide better insights into their aging process. The ACC is a frontal limbic brain region that serves as a key hub connecting cognitive and affective networks^22,23^. This region is characterized by specialized von Economo neurons, which support social-emotional and self-related functions^24^. In our study, increased ACC glucose metabolism in *APOE3^Ch^* carriers relative to non-carriers was associated with greater global cognitive performance, better episodic memory, lower subjective memory complaints and lower burden of depressive symptoms. This suggests that the preservation of glucose metabolism in the ACC may underlie the preservation of cognitive-behavioral function. While we cannot draw causal conclusions in our study, higher glucose metabolism has been causally linked to improved cognitive performance in animal models and genetic human studies^25-27^. Further exploring the functional and structural role of the ACC in this population may shed light on cognitive reserve and aging-resilience mechanisms, which could promote healthy aging.

Of note, brain metabolic as well as cognitive protection in *APOE3^Ch^* carriers was age-dependent, as baseline cerebral glucose metabolism and cognitive scores were lower in *APOE3^Ch^* carriers than non-carriers at younger ages. One possible explanation is the lower years of education and higher GDS scores in *APOE3^Ch^* carriers compared to non-carriers, which could influence cerebral glucose metabolism through brain reserve remodeling^28,29^. Lower baseline cerebral glucose metabolism could also stem from compensatory mechanisms necessary to sustain metabolic function over time, such as neurovascular or neuroinflammation function^30^. More studies are needed to understand cell-specific metabolic protection processes, specifically astrocytic and microglia alterations as well as changes in lipid metabolism pathways. In the UK Biobank sample, *APOE3^Ch^* carriers showed lower polygenic risk for AD compared to non-carriers, even though the *APOE3^Ch^* variant is not included in genome-wide association studies^13^. This finding suggests that epistatic interactions between *APOE3^Ch^* and other genetic variants may contribute to the protection observed.

Several limitations in our study should be noted. While the high prevalence of *APOE3^Ch^* in the Colombian kindred is the key strength of our study, the relatively small sample of *APOE3^Ch^* carriers does not allow for complex statistical modeling and multiple comparisons or stratification into sex and *APOE* genotype. *APOE4* alleles are linked to cerebral glucose hypometabolism, while *APOE2* alleles are associated with preservation of metabolism^31,32^. The cross-sectional and observational design of this study limits inferences about disease progression and aging trajectories and precludes causal interpretations of *APOE3^Ch^* effects. Future biomarker studies in the Colombian kindred will focus on systemic and central vascular changes. Animal models and genetic studies are further necessary to complement our findings with mechanistic insight.

Overall, we present novel evidence consistent with a metabolic resilience phenotype conferred by the *APOE3^Ch^* variant across AD-related pathology as well as in the disease-free adult lifespan. Brain metabolic protection and corresponding benefits in cognitive function may contribute to cognitive reserve through compensatory mechanisms. Understanding the pathways through which *APOE3^Ch^* confers protective effects could inform novel therapeutic avenues to enhance resilience to AD and promote healthy brain aging.

## Methods

### Study participants

In the Colombian kindred, our group has identified a total of 121 *APOE3^Ch^* carriers, among whom 1 was homozygous for *APOE3^Ch^* and heterozygous for *PSEN1^E280A^*, 27 were heterozygous for both *APOE3^Ch^* and *PSEN1^E280A^* variants and 93 were heterozygous for *APOE3^Ch^* but were not *PSEN1^E280A^* carriers^6^. The determination of the *APOE3^Ch^* and *PSEN1^E280A^* status has been described previously^5,6^. Of these, 7 *APOE3^Ch^*-*PSEN1^E280A^* carriers and 33 *APOE3^Ch^* carriers underwent FDG-PET imaging and cognitive examinations. To study *APOE3^Ch^*-related alterations in the adult lifespan (aim 2), we excluded 4 cognitively impaired *APOE3^Ch^* carriers. Clinical status was assessed using the Functional Assessment Staging Tool (FAST), with scores of FAST > 3 indicating objective cognitive impairment^33^. In this study, we additionally included 48 typical *PSEN1^E280A^* carriers and 43 non-carriers for *PSEN1^E280A^* without the *APOE3^Ch^* variant who had available clinical assessments and FDG-PET data, to serve as control groups for each aim, respectively (**Supplementary Fig. 2**). All participants or their representatives provided written informed consent for all study procedures. Clinical and neuroimaging assessments were conducted by trained staff blinded to the genetic status at the Grupo de Neurociencias de Antioquia, Universidad de Antioquia, Medellín, Colombia and the Hospital Pablo Tobón Uribe, Medellín, Colombia. This study was approved by the Universidad de Antioquia institutional review board.

### Clinical assessments

A comprehensive battery of neuropsychological assessments adapted to this Colombian population was conducted in Spanish. In our study, the primary cognitive performance assessments were selected for their sensitivity to detecting early changes in typical *PSEN1^E280A^* carriers across the different cognitive-behavioral domains^34^. Specifically, we included the Mini-Mental State Examination (MMSE, range: 0–30) to assess global cognition^35^; the Consortium to Establish a Registry for Alzheimer’s Disease (CERAD) Word List Learning to assess delayed recall (range: 0–10)^36^; the self-reported Memory Complaint Scale to assess subjective memory complaints (SMC, range: 0–45)^37^; and the Geriatric Depression Scale (GDS, range: 0–15) to assess burden of depressive symptoms^38^. To complement these domains, secondary and exploratory cognitive assessments included the CERAD Boston Naming Test (range: 0–15) to assess language and word retrieval abilities; the Trail Making Parts A and B (TMTA and TMTB) times to assess processing speed and attention as well as executive function, respectively; and Raven’s Matrices (range: 0–15) to assess abstract reasoning^34^.

### Neuroimaging acquisition and processing

FDG-PET scans were acquired on a 64-section PET/computed tomography (CT) scanner (Biograph mCT; Siemens), within a median (Q1,Q3) of 2.7 (0.2, 7.1) months from neuropsychological assessment. Intravenous injection of 5 mCi of FDG was administered in fasting conditions followed by a 30-minute radiotracer uptake. A dynamic emission scan of six 5-minute frames (matrix: 400x400x81, voxel size: 1x1x2 mm^3^) was reconstructed with CT attenuation correction. Frames were aligned, averaged and spatially normalized using affine transformations to a subject-specific group template in the Montreal Neurological Institute (MNI) reference space (isotropic 2 mm^3^ voxel size). The inverse transformation was applied to the Automated Anatomical Labeling 3 (AAL3) atlas to align with each subject’s FDG-PET native space^39^. All images were visually inspected to ensure proper alignment. Next, FDG uptake was normalized to that of the pons, a widely used reference region to minimize intra-subject variability in FDG-PET^40^. The resulting standardized uptake value ratio (SUVR) maps were finally smoothed with a Gaussian kernel of 8 mm full width at half maximum. The mean SUVR was computed for bilateral regions of interest (ROI) of the AAL3 atlas (90 regions). We selected *a priori* the precuneus and the hippocampus as ROIs, considering the regional metabolic changes that occur in AD and aging^9,14^. In addition to ROI-based analysis, SUVR maps in the MNI space were computed to allow for exploratory voxelwise analysis. This approach informed post-hoc analyses of other ROIs.

Magnetic resonance imaging (MRI) was acquired on a 3T Tim Trio (Siemens). The structural T1-weighted image (3D MPRAGE sequence, repetition time: 2300 ms, echo time: 2 ms, inversion time: 900 s, flip angle: 8°, matrix: 192x256x256, voxel size: 0.9x0.9x0.9 mm^3^) was normalized to the MNI template space using non-linear transformation. The inverse deformation field was applied to the AAL3 atlas to obtain bilateral regional volumes in the same ROIs.

### Statistical analysis

Study characteristics were compared between *APOE3^Ch^*-*PSEN1^E280A^* carriers and typical *PSEN1^E280A^* carriers (aim 1) and between non-*PSEN1^E280A^APOE3^Ch^* carriers and non-carriers (aim 2) using Wilcoxon rank sum test and Pearson's χ^2^ test as appropriate. We compared age-related alterations in cerebral glucose metabolism and cognitive performance between groups within each aim. For this purpose, we used robust linear regression models using an interaction term between age and group as main predictor. To explore cerebral glucose metabolism, precuneus or hippocampus FDG SUVR were used as outcome. A similar model was computed at the voxelwise level, after threshold-free enhancement correction and false discovery rate (FDR) correction. To explore cognitive performance, MMSE, CERAD Word List delayed recall, SMC and GDS scores were used as outcomes. For statistically significant interaction effects, which indicate slope differences between groups, we further estimated marginal trends to obtain the outcome-age correlation for each group separately. Robust regression was chosen because it is less sensitive to departures from model assumptions and reduces the influence of outlying observations compared to ordinary least squares regression. Models of FDG SUVR and cognitive performance were all adjusted for age, sex (female/male) and years of education. A post-hoc FDG SUVR model was additionally adjusted for regional volume and GDS scores.

Within aim 2, we transformed the variables of interest into age-adjusted z-scores for *APOE3^Ch^* carriers relative to non-carriers. Positive z-scores indicated higher values for *APOE3^Ch^* carriers than non-carriers, while negative z-scores indicated lower values for *APOE3^Ch^* carriers than non-carriers. The association between FDG SUVR z-scores and cognitive metrics z-scores was computed using robust linear regression adjusted for age, sex and years of education. Exploratory analyses investigated age-related alterations in secondary cognitive metrics as well as their correlations with FDG SUVR. Due to the relatively small sample size of *APOE3^Ch^*-*PSEN1^E280A^* carriers, this analysis was not computed for aim 1. Across the study, we report regression coefficients (β), 95% confidence intervals (CI) and *p*-values. Statistical significance was set at α < 0.05. All analyses were performed using R version 2024.04.2 + 764.

## Supplementary Material

This is a list of supplementary files associated with this preprint. Click to download.

• APOE3ChFDGSupplementaryFinal.docx

## Figures and Tables

**Figure 1:**
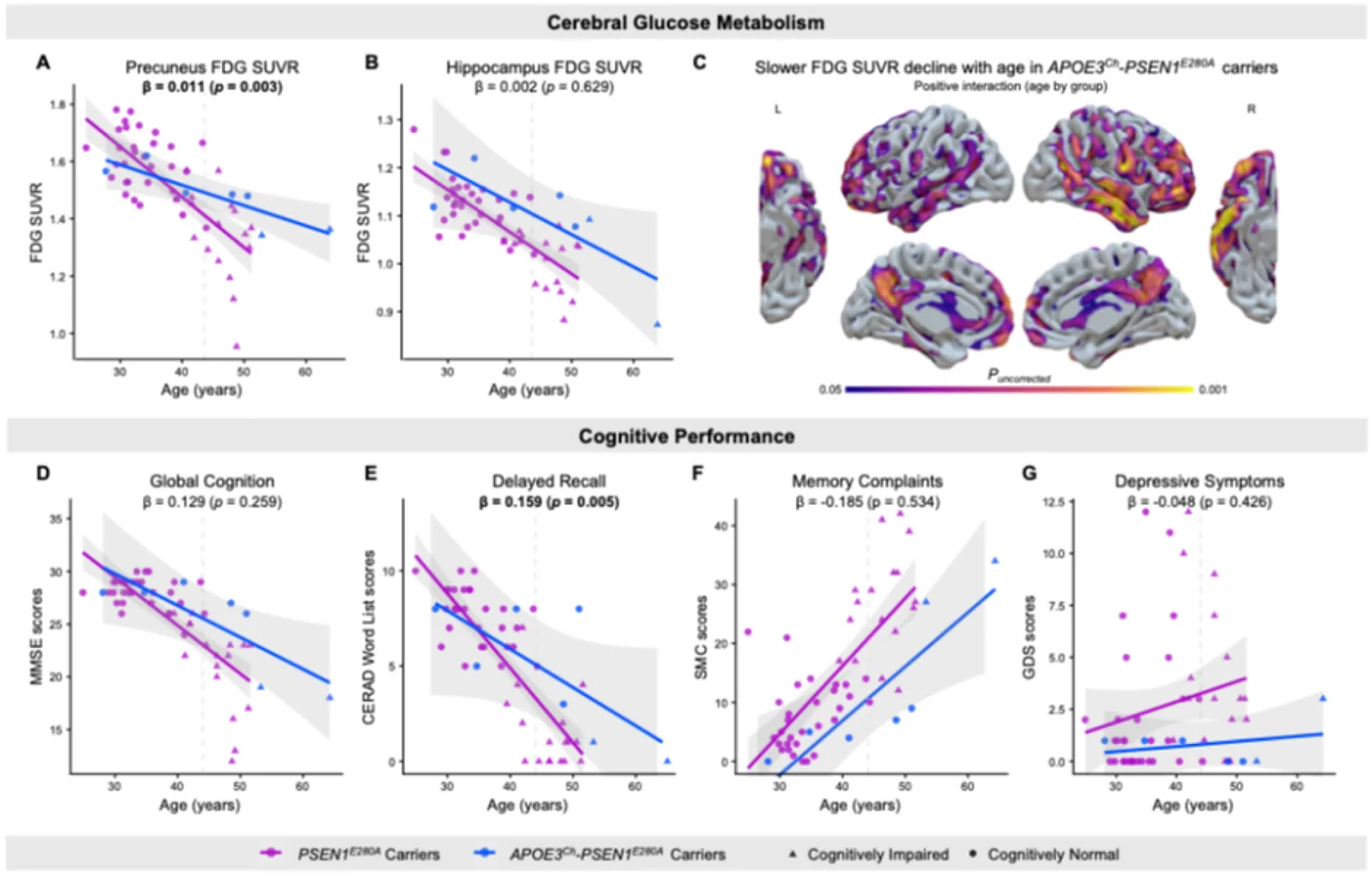
Age-related alterations in cerebral glucose metabolism and cognitive performance in *APOE3*^*Ch*^-*PSEN1*^*E280A*^ carriers compared to *PSEN1*^*E280A*^ carriers across ADAD. Scatterplots showing the correlations between age and precuneus (**A**) and hippocampus FDG SUVR (**B**) per group. FDR-uncorrected voxelwise maps of the slope differences in age-related FDG SUVR changes between groups (**C**). Warm colors represent larger effect sizes for positive interaction effects, representing smaller age-related reduction in FDG SUVR in *APOE3*^*Ch*^-*PSEN1*^*E280A*^ carriers relative to typical *PSEN1*^*E280A*^ carriers. No clusters survived negative interactions. Scatterplots showing the correlations between age and MMSE (**D**), CERAD Word List delayed recall (**E**), SMC (**F**) and GDS (**G**) scores between groups. β represent the regression coefficient for interaction effects, i.e., slope differences between groups, from age, sex and education-adjusted robust regression models. The dashed vertical line represents the estimated age of MCI onset of typical *PSEN1*^*E280A*^ carriers from the Colombian kindred.

**Figure 2:**
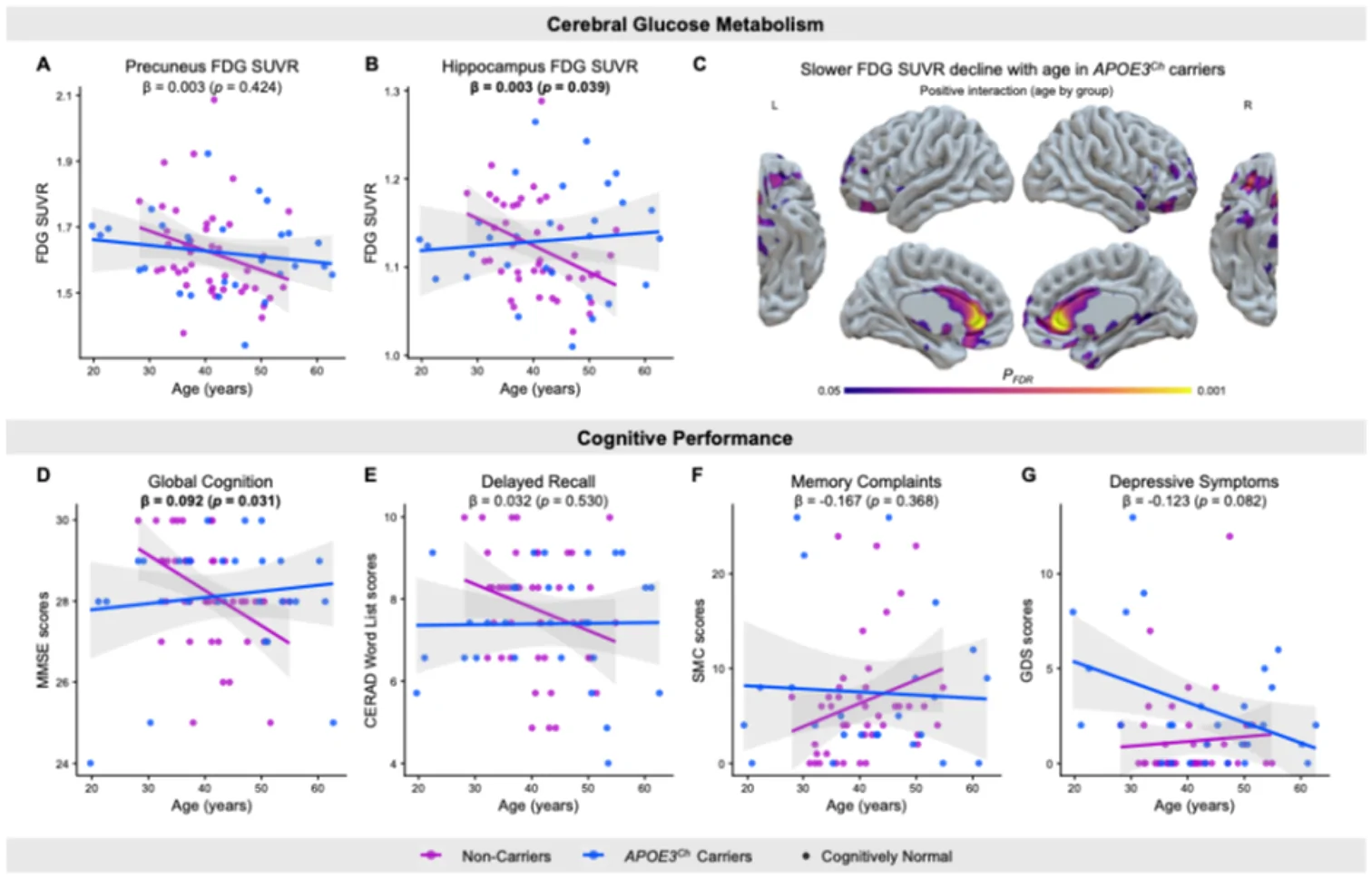
Age-related alterations in cerebral glucose metabolism and cognitive performance in cognitively unimpaired *APOE3*^*Ch*^ carriers compared to non-carriers across the adult lifespan. Scatterplots showing the correlations between age and precuneus (**A**) and hippocampus FDG SUVR (**B**) per group. FDR-corrected voxelwise maps of the slope differences in age-related FDG SUVR changes between groups (**C**). Warm colors represent larger effect sizes for positive interaction effects, representing smaller age-related reduction in FDG SUVR in *APOE3*^*Ch*^ carriers relative to non-carriers. No clusters survived negative interactions. Scatterplots showing the correlations between age and MMSE (**D**), CERAD Word List delayed recall (**E**), SMC (**F**) and GDS (**G**) scores between groups. β represent the regression coefficient for interaction effects, i.e., slope differences between groups, from age, sex and education-adjusted robust regression models.

**Figure 3:**
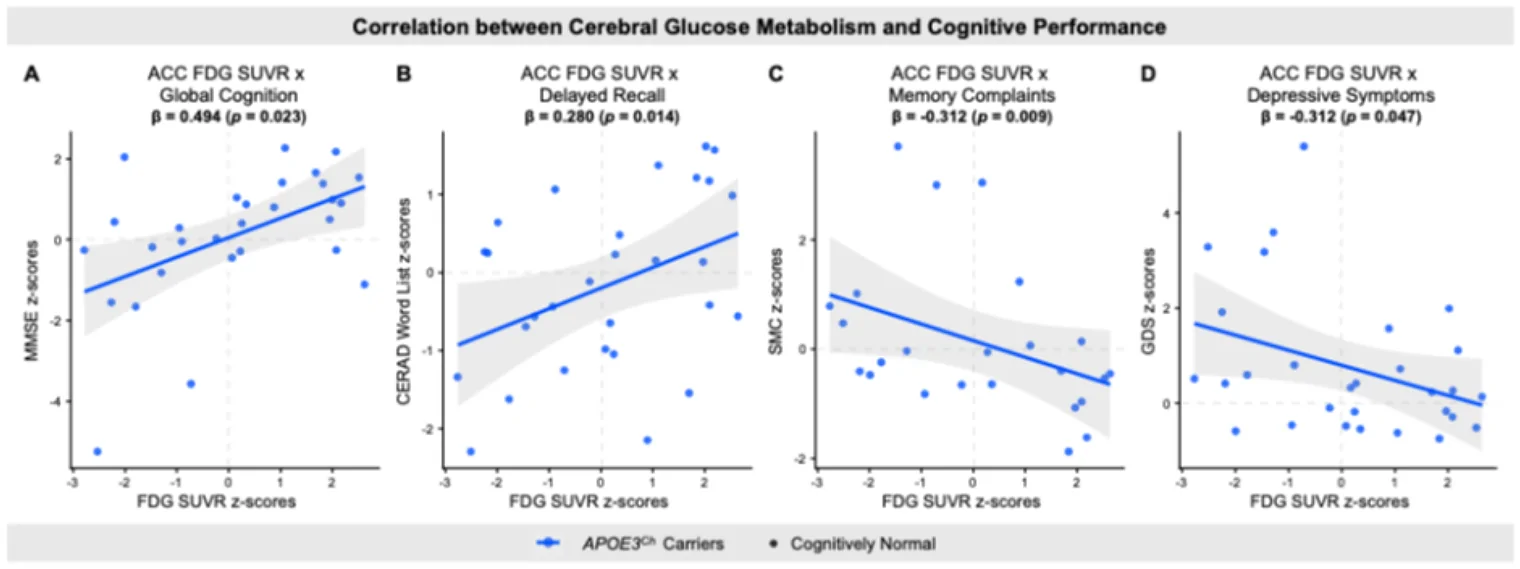
Associations between cerebral glucose metabolism and cognitive performance in cognitively unimpaired *APOE3*^*Ch*^ carriers across the adult lifespan. Scatterplots showing the correlation between FDG SUVR z-scores in the anterior cingulate cortex (ACC) and MMSE (**A**), CERAD Word List delayed recall (**B**), SMC (**C**) and GDS (**D**) age-adjusted z-scores in *APOE3*^*Ch*^ carriers relative to non-carriers. Dashed horizontal and vertical lines represent the quadrants for positive and negative age-adjusted z-scores, with positive z-scores representing higher values for *APOE3*^*Ch*^ carriers than non-carriers. β represent the regression coefficient for the main effect from age, sex and education-adjusted robust regression models.

**Table 1.** Sample demographics by group.

	*_PSEN1_E280A* Carriers N = 48^1^	*APOE3^Ch^*- *PSEN1^E280A^* Carriers N = 7^1^	p-value^2^	Non-Carriers N = 43^1^	*APOE3^Ch^* Carriers N = 29^1^	p-value^2^
Age (years)	39 (32, 45)	49 (35, 53)	0.10	40 (36, 44)	43 (35, 50)	0.3
Female sex (n)	30 (63%)	2 (29%)	0.12	26 (60%)	15 (52%)	0.5
Education (years)	11 (7, 13)	11 (7, 11)	0.2	12 (8, 15)	11 (5, 13)	**0.034**
Cognitively impaired (n)	17 (35%)	2 (29%)	>0.9	0 (0%)	0 (0%)	>0.9
MMSE score	27 (23, 29)	27 (19, 28)	0.7	28 (28, 29)	28 (28, 29)	>0.9
CERAD WLL delayed recall score	7 (2, 8)	5 (1, 8)	0.5	8 (6, 9)	7 (6, 8)	0.3
Naming score	13 (12, 14)	13 (12, 15)	0.7	13 (12, 14)	13 (11, 14)	0.4
TMTA time (s)	45 (36, 85)^†^	63 (39, 70)^†^	0.8	40 (30, 50)	49 (39, 62)	**0.016**
TMTB time (s)	94 (55, 164)^†^	108 (58, 258)^†^	0.8	80 (51, 119)^†^	107 (74, 151)^†^	0.077
Raven score	10 (7, 10)^†^	10 (8, 11)	0.6	10 (8, 11)	10 (9, 11)	0.4
SMC score	12 (5, 24)^†^	7 (4, 27)	0.5	6 (2, 8)^†^	5 (3, 9)^†^	0.9
GDS score	1.0 (0, 3.5)	1.0 (0, 1.0)	0.2	0 (0, 2)	2 (1, 4)	**0.002**

1 Median (Q1, Q3); n / N (%)

2 Wilcoxon rank sum test; Pearson's χ^2^ test

† Missing values in *PSEN1^E280A^* carriers for TMTA time (n=1), TMTB time (n=19), abstract reasoningscore (n=1) and subjective memory complaints score (n=1); in *APOE3^Ch^* + *PSEN1^E280A^* carriers forTMTA time (n=1) and TMTB time (n=3); in non-carriers for TMTB time (n=8) and subjective memorycomplaints score (n=1); and in *APOE3^Ch^* carriers for TMTB time (n=8) and subjective memorycomplaints score (n=5)

## Data Availability

Data are not publicly available due to privacy or ethical restrictions. Anonymized clinical, genetic and imaging data supporting the findings of this study are available upon request, subject to an internal review by the corresponding author to ensure that anonymity, confidentiality, and genetic status are protected. Data requests will be considered based on a proposal review, and completion of a data sharing agreement, in accordance with the University of Antioquia.
